# A prospective study to investigate the role of serial serum mesothelin in monitoring mesothelioma

**DOI:** 10.1186/s12885-018-4113-3

**Published:** 2018-02-17

**Authors:** Duneesha de Fonseka, David T. Arnold, Louise Stadon, Anna Morley, Emma Keenan, Michael Darby, Lynne Armstrong, Paul Virgo, Nick A. Maskell

**Affiliations:** 1 Academic Respiratory Unit, School of Clinical Sciences, University of Bristol, Learning and Research Centre, Southmead Hospital, Westbury-on-Trym, Bristol, BS10 5NB UK; 2 Department of Radiology, Southmead Hospital, North Bristol NHS Trust, Bristol, BS10 5NB UK; 3 Department of Radiology, University Hospitals Bristol, Bristol, BS2 8HW UK

**Keywords:** Mesothelioma, Mesothelin, Biomarkers, Monitoring

## Abstract

**Background:**

Radiological monitoring of malignant pleural mesothelioma (MPM) using modified RECIST criteria is limited by low sensitivity and inter-observer variability. Serial serum mesothelin measurement has shown utility in the assessment of treatment response during chemotherapy but has never been assessed in the longer term follow up of patients.

**Methods:**

This is a single centre study of consecutive patients diagnosed with MPM who received chemotherapy or best supportive care (BSC). Serum mesothelin measurements with paired 6 monthly CT scans were performed following the completion of chemotherapy, or from baseline in the BSC group. Changes in mesothelin were correlated with radiological progression and overall survival.

**Results:**

Forty-one patients with MPM were recruited and followed up for a minimum of 12 months (range 12–21 months). The majority of patients (*n* = 23) received chemotherapy with pemetrexed and cisplatin. Across the cohort a 10% rise in serum mesothelin could predict radiological progression with a sensitivity of 96% (IQR; 79–100) and specificity of 74% (IQR; 50–91). Sensitivity fell to 80% in sarcomatoid only disease. Patients with a rising mesothelin at 6 months had significantly worse overall survival (175 days) compared to stable/falling levels (448 days) (*p* = 0.003).

**Conclusions:**

This is the first study to assess serum mesothelin’s ability to detect progression of MPM following chemotherapy or during BSC. A 10% rise in serum mesothelin level showed excellent sensitivity at predicting progressive disease. Mesothelin measurement has several advantages over serial CT imaging including reducing hospital visits and cost.

## Background

Malignant pleural mesothelioma (MPM) is an aggressive malignancy with a median survival of 9–14 months [1]. There has been little improvement in overall survival from the disease over the last decade with limited development of effective treatments. Chemotherapy is the only treatment modality that has shown to improve survival following the results of a large randomised control trial (RCT) of pemetrexed and cisplatin which conferred an additional 2 months to overall survival compared to cisplatin alone [2]. Due to a response rate of only 25% and a significant side effect profile many patients decline or are unsuitable for first line chemotherapy [3]. The role of non-chemotherapeutic options such as biological treatments or immunotherapy is the topic of many ongoing clinical trials [4–8]. Additionally, the efficacy of second line chemotherapy in patients who have progressed following standard chemotherapy is uncertain with promising reports from the use of vinorelbine, gemcitabine or re-challenging with pemetrexed [9–12].

Given the low response rate to conventional treatment, and uncertainty around the efficacy of newer therapies, the ability to accurately monitor disease is essential for oncologists and trialists. The current standard for monitoring is, as for many tumours, serial computed tomography (CT) scans. However, unlike many tumours, MPM does not grow spherically, instead growing as a ‘rind’ encasing the thoracic cavity. An adaptation of the Response Evaluation Criteria in Solid Tumours (RECIST) to the modified RECIST criteria has only partially allowed for this unique morphology. Other issues including low sensitivity to predict progression, subjectivity in radiologists’ choice of measurement sites and complications from the presence of pleural plaques or fluid, mean research into a more robust and reproducible radiological marker is ongoing [13]. Studies have shown that serial FDG-PET (Fludeoxyglucose positron emission tomography) scanning can be used to predict response [14] but is significantly limited by false positive uptake in inflamed tissue from surgical intervention or pleurodesis, as well as restricted availability or limited reimbursement from insurers [15].

Serum or pleural fluid biomarkers that could reflect disease activity are an attractive option for MPM given the difficulties with radiological assessment. The most investigated is mesothelin, a membrane-bound glycoprotein overexpressed by malignant mesothelial cells [16]. Soluble mesothelin is found in the blood and pleural fluid of patients with MPM and levels correlate with tumour stage and bulk. It lacks the sensitivity to be used as a diagnostic marker given reduced expression in non-epithelioid MPM. There remains uncertainty as to whether the baseline mesothelin level is a useful predictor of prognosis. However, a systematic review we performed found 8 studies that had assessed serum mesothelin’s ability to monitor disease during chemotherapy and found it correlated with radiological markers and survival [17]. No studies had assessed the utility of mesothelin in longer term monitoring patients not currently receiving chemotherapy. In this prospective cohort study, we aim to assess the ability of serum mesothelin to monitor disease at later timepoints in patients who have completed chemotherapy or those receiving best supportive care (BSC).

## Methods

### Patients

From February 2014 to October 2016 consecutive patients were prospectively recruited to a longitudinal study of biomarkers alongside normal clinical care. All patients gave written informed consent and the trial was approved by the South West REC (Ref 08/H0102/11). Eligibility criteria included; histocytologically confirmed malignant pleural mesothelioma, age over 18 years and an expected life expectancy of greater than 3 months. At baseline, patient demographics, method of diagnosis of MPM and WHO performance status were recorded. Blood samples were taken at baseline and a full staging CT scan was performed.

This study had no impact on the treatments received by the patient, but treatment information was recorded.

For eligible patients, chemotherapy was given in the form of up to 6 cycles of Pemetrexed and Cisplatin or Carboplatin (as tolerated by the patient).

### CT imaging

All patients had a baseline full staging CT scan. Patients who received chemotherapy had a CT scan at 3, 6 and 12 months to assess recurrence. Patients in the non-chemotherapy group had a CT scan at clinically appropriate intervals (usually every 6 months). CT scans were reported by two methods. Firstly, they were all reported in a standard reporting fashion for clinical purposes by a consultant thoracic radiologist who classified the scans into radiological progression, stability or partial response but did not use a formal reporting criteria (herein described as the ‘clinically reported CT’). Secondly, another independent radiologist assessed the imaging using the modified RECIST criteria (herein described as the ‘mRECIST CT’). The mRECIST criteria defines complete response as the disappearance of all target lesions with no visible tumour elsewhere and partial response (PR) as at least a 30% reduction in cumulative tumour measurement. Progressive disease (PD) is defined as an increase of 20% in cumulative tumour measurement or emergence of new lesions. Stable disease (SD) is defined as not fulfilling the criteria for PR of PD. Both radiologists were blinded to mesothelin results.

### Mesothelin measurement

Serum samples for mesothelin testing were collected at respiratory or oncology clinic appointments. They were analysed routinely using the commercial Mesomark™ ELISA (Fujirebio) using manufacturer’s recommended methods for sample processing, by investigators unware of the patients clinical or radiological characteristics. Baseline blood tests of liver and renal function were collected but not performed again unless clinically indicated.

### Statistical analysis

Mesothelin levels are not normally distributed so results are presented as medians with interquartile ranges (IQR). Analysis of baseline levels of mesothelin between groups was performed using the Mann Whitney U test, with a *p* value of less than 0.05 considered statistically significant. Cox regression analysis was performed to determine whether baseline mesothelin, as both a continuous and bivariate variable, impacted on survival.

The ability of serum mesothelin to predict radiological progression was assessed using timepoint analysis. A mesothelin measurement with a paired CT scan (defined as performed within a maximum of 31 days of each other) was called a ‘timepoint’. These timepoints were grouped depending on the time from baseline into 3 monthly intervals. Two timepoints in the same patient allowed for a ‘comparison’ between the percentage change in mesothelin level and the radiological report (both clinically reported and mRECIST CTs). Patients were grouped into whether their CT scan showed progression of disease or stable disease/partial response. This study aimed to assess the role of mesothelin in patients not currently undergoing treatment, so any timepoints during or within 1 month of chemotherapy were not included. Additionally, for any patients who had previously received chemotherapy, the post-chemotherapy mesothelin and CT scan was considered the new point of reference, not the pre-chemotherapy baseline.

For the timepoint analysis a change in mesothelin was defined as a relative change from the previous mesothelin level, either ‘falling’ or ‘rising’, with separate analyses performed using 10%, 15% and 25% cut-offs. The thresholds were calculated by the following method; ((Later timepoint mesothelin – Earlier timepoint mesothelin)/Earlier timepoint mesothelin) * 100. The ability of mesothelin to predict radiological progression was assessed using sensitivity, specificity, predictive values and accuracy. Exploratory subgroup analysis was carried out based on baseline mesothelin level and histological type.

To further assess the ability of changes in mesothelin to track disease, independent of radiological assessments, the impact of a rising level on survival was calculated and compared to stable mesothelin levels and other poor prognostic indicators.

## Results

### Participants

In total, 41 patients with malignant pleural mesothelioma were recruited to this study and had a mesothelin taken at baseline alongside usual care (see Table 1). The majority were males (35 vs 6) and the cohort had a median age of 72 (range 58–83). Twenty-three patients received chemotherapy in the form of Pemetrexed and Cisplatin, with no patients being referred for surgical intervention. Eighteen patients received no chemotherapy either due to choice, poor performance status, or intention for delayed chemotherapy by the physician. Patients in the non-chemotherapy group were older on average (75 vs 69) but had a similar distribution of histological subtypes. Three patients in the cohort were diagnosed on the basis of pleural fluid cytology, therefore histological subtype was unknown.

**Table 1:** Baseline demographics, mesothelins and survival

	Post-chemotherapy	Non-chemotherapy
Number	23	18
Median age (range)	69 (58–77)	75 (67–83)
Male/Female	22/1	13/5
Histology		
Epithelioid	14	11
Biphasic	3	1
Sarcomatoid	5	4
Unknown	1	2
Baseline mesothelin		
Median	1.9	3.4
(IQR)	(1.2–13.4)	(1.2–6.4)
Survival (months)		
Median	17	10
(IQR)	(14–26)	(7–22)

### Baseline mesothelin

All 41 patients had a serum mesothelin performed at baseline, with a median of 2.3 nmol/L (IQR 1.2–6.6) across the entire cohort. Baseline mesothelin was significantly higher in the epithelioid (3.1 nmol/L IQR 1.25–13.6) compared to non-epithelioid (1.3 nmol/L IQR 0.75–2.95) histological subtypes (*p* = 0.041). Survival of the entire cohort was 15 months (IQR 10–23). When analysed as a bivariate variable (dichotomizing the cohort at a mesothelin level of 2 nmol/) there was no difference in survival between those with a low (< 2 nmol/L) or high (> = 2 nmol/L) baseline mesothelin with median survival times of 329 (IQR 225 to 587) and 452 days (IQR 310 to 777) respectively (*p* = 0.191). Additionally, baseline mesothelin did not impact on survival when analysed as a continuous variable (*p* = 0.193). There was no significant difference on baseline mesothelin or change in mesothelin levels depending on the patients’ baseline renal function (eGFR).

### Change in mesothelin and radiological progression

At baseline, from the cohort of 41 patients, all had a CT scan within 31 days of a serum mesothelin measurement for comparison to later timepoints. The numbers of other timepoints were; 5 at 3 months, 21 at 6 months, 4 at 9 months, 15 at 12 months, and 6 at later timepoints. This allowed for 43 comparisons, with 25 in the post-chemo group and 18 in the non-chemotherapy group. The median time between mesothelin measurement and CT was 13 days (range 0 to 31 days).

When all comparisons were amalgamated across the cohort there was no change in median mesothelin levels in patients who had not progressed on the clinically reported CT (0.0 nmol/l (IQR -0.6 – 0.4)). This compared to a rise of 1.8 nmol/l (IQR 0.7–1.8) in patients with radiological progression of disease (*p* < 0.001).

**Fig. 1:**
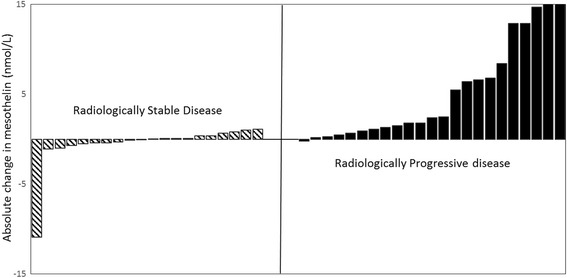
Waterfall plot to show change in mesothelin between Progressive disease and Stable/Partial disease

Results from Table 2 demonstrated the ability of a rising mesothelin to predict radiological progression on both clinically reported and mRECIST CT in individual patients. A variety of pre-defined cut-offs were tested. Regardless of cut-off used, mesothelin tracked the clinically reported CT results with greater accuracy than mRECIST CT reports. Resultantly, the clinically reported CT has been used for the ongoing analysis. Figure 1 is a waterfall plot of the absolute changes of mesothelin divided by clinically reported CT report (SD and PR versus PD). It demonstrated that the vast majority of patients with radiologically progressive disease had a rising mesothelin (with only 1 patient having a small fall in level).

**Table 2:** Ability of mesothelin to predict radiological progression depending on cut-off used and radiological reporting method

Mesothelin Cut-off	10%		15%		25%	
CT reporting method	Clinically reported CT	MRECIST	Clinically reported CT	MRECIST	Clinically reported CT	MRECIST
No.	43	25	43	25	43	25
Sensitivity	95.8 (78.8–99.8)	90.9 (58.7–99.7)	83.3 (62.6–95.3)	72.7 (39.0–93.9)	80.0 (59.3–93.2)	72.7 (39.0–93.9)
Specificity	73.7 (48.8–90.8)	57.1 (28.9–82.3)	84.2 (60.4–96.6)	71.4 (41.9–91.6)	84.2 (60.4–96.6)	71.4 (41.9–91.6)
PPV	82.1 (68.3–90.8)	62.5 (46.9–75.8)	87.0 (69.9–95.0)	66.7 (44.7–83.2)	87.0 (69.9–95.0)	66.7 (44.7–83.2)
NPV	93.3 (66.8–99.0)	88.9 (53.9–98.2)	80 (61.7–90.9)	76.9 (54.6–90.2)	76.2 (58.8–87.8)	76.9 (54.6–90.2)
Accuracy	86.0%		83.7%		0.81%

Further subgroup analysis was performed depending on baseline mesothelin or histological subtype, see Table 3. Serial changes in mesothelin predicted radiological progression with 100% sensitivity in patients with a high baseline mesothelin (defined as > 2 nmol/l). There was a fall in sensitivity and specificity in patients with low baseline mesothelin or sarcomatoid disease, although sensitivity remained above 90% in the former group.

**Table 3:** Ability of mesothelin to predict progression- subgroup analysis

	Baseline Mesothelin> = 2	Baseline Mesothelin< 2	Non-Epithelioid
*n*	19	24	9
Progression on CT (Y/N)	13/6	11/13	5/4
Sensitivity	100 (75.3–100)	90.9 (58.7–99.7)	80.0 (28.4–99.5)
Specificity	83.3 (35.9–99.6)	69.2 (38.5–90.9)	75.0 (19.4–99.4)
PPV	92.7 (68.4–98.7)	71.4 (51.9–85.2)	80.0 (40.9–95.9)
NPV	100	90.0 (57.3–98.4)	75.0 (32.2–94.9)

### Change in mesothelin and overall survival

At 6 months, patients with a rising mesothelin (using a 10% cut-off) had a significantly shorter survival (from the date of sampling) compared to those with a falling or stable level (*p* = 0.003). Those with a rising mesothelin had median survival of 175 days (IQR 80–211) compared to 448 days (IQR 321 to 554). Patients with radiologically progressive disease on clinically reported CT had a median survival of 170 days (IQR 72–223) from the date of CT scan compared to 433 days (IQR 255–581) in those with stable disease or partial response (*p* = 0.001). The majority of CT scans (10/21) performed at a 6 month time point were non-measurable using mRECIST criteria so these results were not analyzable. In a multivariate analysis including age (≥70 vs < 70), histological subtype (epithelioid vs non-epithelioid) and treatment modality neither change in mesothelin or clinically reported CT remained significant.

## Discussion

This study assessed the ability of serum mesothelin to detect progression of MPM in patients following chemotherapy or receiving best supportive care. To our knowledge this is the first study to focus on longer term monitoring as opposed to mesothelin’s role as a marker of treatment response during or immediately after chemotherapy. The ability to monitor MPM in these patients is becoming increasingly important. However, no specific evidence exists for how patients with MPM should be monitored following first line chemotherapy. Recent guidelines have suggested that 3–4 month follow up is good practice without specifying what form this follow up should take https://www.brit-thoracic.org.uk/standards-of-care/guidelines/bts-statement-on-malignant-mesothelioma/. There is considerable variation in practice nationally, for two main reasons. Firstly, until recently many oncologists felt there was no data to support the role of second line chemotherapy in this setting. In addition, there was limited access to clinical trials after first line treatment. There was, therefore, no perceived benefit from monitoring patients post first line chemotherapy or in those having BSC. However, given promising results from second line chemotherapy trials, as well as non-chemotherapeutic options this perception is changing. Secondly, no radiological marker has shown the ability to accurately monitor disease. A serum biomarker that could identify disease progression would be very useful to oncologists and physicians, with the additional benefit of reducing patient burden and healthcare costs. Mesothelin is the most studied serum biomarker for mesothelioma with the majority of literature assessing its diagnostic potential. Although a raised level is fairly specific for MPM, many patients with non-epithelioid disease will have unrecordable levels even at an advanced stage. This has limited its utility as a diagnostic test, although recent studies have attempted to combine it with more novel markers [18, 19]. The role of biomarkers in other malignancies has often begun as a putative diagnostic marker before becoming a marker of treatment response or recurrence (CA125, PSA).

Previous literature has shown that mesothelin can be used as a marker of treatment response when measured serially. The first example from Grigoriu and colleagues [20] took patients with a positive (> 1 nM/L) mesothelin at baseline receiving chemotherapy and immunotherapy (*n* = 40). They found that in patients with a 10% or greater rise in mesothelin there was a 75% chance of radiological progression using mRECIST as well significantly worse overall survival. The largest study of its type [21] correlated both CT scans (mRECIST) and PET (TGV and tumour volume) with change in mesothelin levels after treatment (using a 25% cut-off). In the chemotherapy group (*n* = 55) the change in mesothelin significantly correlated with radiological response on CT (*p* = 0.023). Although only a minority of the cohort (*n* = 28) could be reassessed using PET imaging, due to prior pleurodesis or surgery, the change in both metabolic activity (TGV) and tumour bulk strongly correlated with change in mesothelin levels (*p* < 0.001). Survival analysis demonstrated that the trend of mesothelin correlated with survival in a multivariate model that included age, sex, histology and treatment. Interestingly, this was not true when tumour volume on PET was added to the model, indicating that mesothelin was probably acting as a proxy for tumour bulk.

Across the literature we found several different thresholds for defining a clinically significant change in mesothelin level, including 10% [20], 15% [22] and 25% [21]. Wheatley-Price and colleagues [23] performed a post-hoc analysis of mesothelin values from a cohort of 42 patients using absolute (5 mmol) and relative (10%) changes and their correlation with survival, finding that relative changes were more accurate. This current study assessed a variety of cut-offs, finding that a 10% cut-off reduced the specificity of a rising mesothelin level but had a sensitivity of 96%. Given our aim was to assess the ability of mesothelin to detect disease progression we felt this reduction in specificity was justified with a false positive rate of 28% but a false negative rate of only 4%.

We demonstrated that disease progression could be detected using mesothelin with an accuracy of 86% and NPV of 94%. Unlike some previous literature [20, 23] this analysis did not exclude patients from the analysis with low baseline mesothelin or non-epithelioid disease. There has been uncertainty about the utility of serially measuring mesothelin in these patients. In this study, 44% (8/19) of patients with a mesothelin of less than 2 mmol/L at baseline had an increased level (range 2.1–20.4) at later timepoints. Additionally, when the analysis was limited to patients with a baseline mesothelin of < 2 mmol there was only a limited reduction in sensitivity to predict progression. Given the small subgroup, no firm recommendations can be made regarding the utility of retesting mesothelin when the baseline is negative (< 2 nmol/l). This needs further assessment in other prospective studies.

This study had several limitations that could impact on the conclusions drawn. The cohort of patients was small, which has an impact on the conclusions of the multivariate analyses, but comparable to other studies of its type. This was an observational study alongside normal clinical practice and there were instances where a mesothelin was collected but the patient did not receive a scan within the pre-allocated 1 month period. Additionally, given the aggressive nature of MPM very few comparisons of timepoints over 12 months were available, but this is likely to be a pragmatic assessment of following up these patients in clinical practice. As previously mentioned other studies have monitored the change in mesothelin levels in response to systemic therapy. As a result, these studies had a proportion of patients where mesothelin levels fell considerably, which was correlated with radiological ‘partial response’. Given this study focused on patients not receiving treatment there were few instances of a falling mesothelin. Worsening renal function has been shown to falsely elevate mesothelin levels [24]. Although there was no difference in mesothelin sensitivity between patients with normal and abnormal renal function at baseline, there was no ongoing assessment during follow up. Finally, the difficulty with studies of this type is that the current ‘gold standard’ of monitoring (mRECIST CT) which mesothelin trends are compared to has been shown to be insensitive at detecting early progression. This analysis found that mesothelin correlated better with the clinically reported CT scan compared to mRECIST reporting, a finding of other similar studies [23]. Additionally, over half (25/43) of the CT scans used in the timepoint analysis were non-measurable by mRECIST criteria with a short axis diameter of less than 1 cm. This is another shortcoming of the mRECIST criteria that limits its sensitivity and applicability in clinical practice. It is for this reason that we assessed the impact of a changing mesothelin levels on survival as a means of validation, finding that a rising mesothelin at 6 months was a poor prognostic indicator. As with other cancer biomarkers [25] the reason for several ‘false-positive’ results (where a rising mesothelin occurred in the context of radiologically stable disease) could be because changes in mesothelin preceded radiological change.

## Conclusions

We have demonstrated that a rising serum mesothelin is a sensitive marker of progression in the follow up of patients with MPM. Given the emergence of effective non-chemotherapeutic treatments and second-line agents, accurate disease monitoring is becoming increasingly important. This study has demonstrated that regular mesothelins could be used as a cost effective adjunct or alternative to serial CT scanning.

## Abbreviations

BSC: Best supportive care
CT: Computed tomography
FDG-PET: Fludeoxyglucose positron emission tomography
MPM: Malignant pleural mesothelioma
PD: Progressive disease
PR: Partial response
RCT: Randomized controlled trial
RECIST: Response Evaluation Criteria in Solid Tumours
SD: Stable disease
TGV: Total glycolytic volume
